# Olive Leaf Extract Health-Promoting and Anti-Tumor Properties: An Adjunct Therapy in Pediatric Oncology?

**DOI:** 10.3390/nu18111755

**Published:** 2026-05-29

**Authors:** Irma Airoldi, Chiara Brignole, Fabio Morandi

**Affiliations:** 1 UOSD Laboratory of Cell Therapies, IRCCS Istituto Giannina Gaslini, 16147 Genova, Italy; fabiomorandi@gaslini.org; 2 UOSD Laboratory of Experimental Therapies in Oncology, IRCCS Istituto Giannina Gaslini, 16147 Genova, Italy; chiarabrignole@gaslini.org

**Keywords:** OLE, pediatric tumors, nutraceuticals, adjuvant

## Abstract

Modern scientific research increasingly views olive leaf extract (OLE) not merely as a simple supplement, but as a sophisticated chemical orchestra where a wide array of phytochemicals works in natural harmony to provide therapeutic benefits. While olive oil is the most famous product of the *Olea europaea* tree, it is important to emphasize that the leaves are actually a far richer and more concentrated reservoir of bioactive molecules, often containing phenolic levels several times higher than those found in the fruit or oil. This whole plant extract often proves more biologically effective than isolated compounds because its components target multiple cellular pathways simultaneously. Many beneficial effects have been ascribed to OLE including anti-inflammatory, anti-oxidant, anti-microbial, anti-viral, neuroprotective, and anti-tumoral effects. In this review, we focused on the latter activity, especially in the field of pediatric tumors such as acute leukemias and neuroblastoma. This issue was discussed starting from the definition of OLE and its components describing the main biological activities, passing through the OLE roles on the immune system, moving on to the anti-cancer functions, and ending with future perspectives.

## 1. Olive Leaves Extract Components

**Figure 1:**
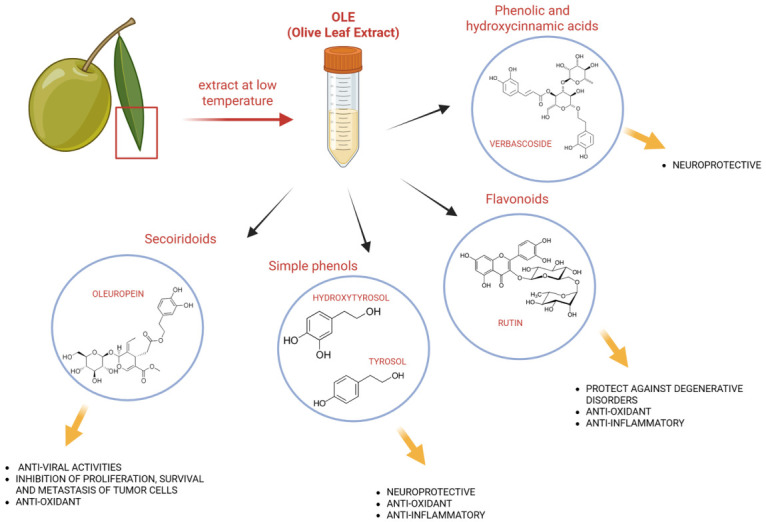
Composition of OLE and principal effects of the different molecules. Created in BioRender. Morandi, F. (2026) https://BioRender.com/9bp2cdo, Accessed on 29 May 2026.

Olive leaf extract (OLE) is a complex matrix rich in bioactive compounds. Its exact composition varies significantly depending on many factors such as the olive tree geographical origin, harvesting time, drying methods, and the solvent used for extraction. In general, OLE is much more concentrated in bioactive molecules than extra-virgin olive oil [1,2,3,4,5,6]. The components of OLE can be categorized into different groups including (i) secoiridoids, (ii) simple phenols, (iii) flavonoids, (iv) phenolic and hydroxycinnamic acids and (v) other bioactive components, as described in Figure 1.

### 1.1. Secoiridoids

Secoiridoids are the most abundant and characteristic class of compounds in OLE. Of these, oleuropein is the primary bioactive component, representing 17% to 25% of the dry weight of the extract. Oleuropein typically ranges from 6% to 9% (60–90 mg/g) in dry leaves, whereas in some ethanolic extracts concentrations varying between 20% and 25% of total dry weight have been recorded [7,8]. Commercial standardized extracts often contain approximately 19.8% oleuropein [9]. Other preparations show concentrations of 174.64 mg/g for the Ravece cultivar [10] or as high as 356 mg/g for the Sevillano variety [11]. Notably, in aqueous extracts, such as those provided by Evergreen Life, the mean concentration is of 2656 mg/L, as reported [12].

Oleuropein is responsible for the extract’s bitter taste and many of its pharmacological properties. As the primary bioactive component, it has been extensively studied and documented for its potent anti-viral activities against a variety of viruses, including Herpes simplex virus 1, Epstein–Barr, Hepatitis B virus, and rotavirus [8,11,13,14]. It has also been described as a pleiotropic molecule that exerts chemo-preventive effects by inhibiting the proliferation, survival, and metastasis of tumor cells, while also promoting bone health by stimulating osteoblasts and preventing osteoporosis [15]. Furthermore, its ability to act as a selective pro-oxidant in cancer cells while providing anti-oxidant protection to healthy ones has been highlighted [16]. Oleuropein aglycone represents an additional potent anti-oxidant and anti-inflammatory derivative of oleuropein. A recent study [17] highlighted its significant neuroprotective properties, reporting that it is capable of crossing the blood–brain barrier and interfering with the aggregation of toxic proteins associated with Alzheimer’s and Parkinson’s diseases. Additional secoiridoids present in OLE are ligstroside and oleoside.

### 1.2. Simple Phenols

They are mainly associated with the OLE’s anti-oxidant properties and include hydroxytyrosol (HT) and tyrosol. HT [9] represents the major bioactive component derived from the hydrolysis of oleuropein during olive ripening and processing, known for its high bioavailability and powerful anti-oxidant capacity. HT has a simple molecular structure, making it easy for the human body to assimilate, with high bioavailability. It is a remarkable amphipathic molecule, meaning it is both water- and fat-soluble, which allows it to cross cellular membranes with ease and circulate effectively throughout the human body [10,11]. In addition, it has been documented that HT is rapidly assimilated by the body, reaching peak plasma levels within just 15 to 20 min, yet it is efficiently cleared by the kidneys within 6 to 8 h, preventing toxic accumulation [12]. The anti-oxidant potential is primarily attributed to the presence of the o-dihydroxyphenyl moiety. Thus, HT may donate hydrogen atoms to peroxyl radicals neutralizing harmful free radicals and stimulating the body’s own internal anti-oxidant enzymes, such as superoxide dismutase and glutathione peroxidase [13], reinforcing the natural defense mechanisms against oxidative stress and activating distinct cellular signaling pathways. Due to its small size, HT may cross the blood–brain barrier, making them vital allies in preventing neurodegeneration and widely regarded as one of the most powerful natural neuroprotective agents. Different studies explored how HT and oleuropein protect neurons from the multi-front assault of protein misfolding and inflammation [8,14]. The authors revealed that in Alzheimer’s disease, HT and oleuropein prevent the toxic clumping of β-amyloid peptides, while in Parkinson’s they may stabilize α-synuclein into non-toxic forms. Furthermore, the authors [8] pointed out that HT can restore proper insulin signaling in the brain, combating the cerebral insulin resistance often associated with cognitive decline.

Tyrosol is another simple phenolic alcohol that is present at around 2.28 mg by 100 g leaf extract. While having a slightly different anti-oxidant profile than HT due to its single hydroxyl group, it remains highly resistant to auto-oxidation and is rapidly absorbed by the human body [11].

### 1.3. Flavonoids

Flavonoids are the essential supporting instruments in the chemical orchestra of OLE. Defined as a class of polyphenolic compounds synthesized by plants as a response to microbial infections [18], flavonoids like luteolin, apigenin, and rutin provide a multi-layered defense system for the human body. These molecules are crucial because they synergistically reinforce the pharmacological actions of other olive phenols, targeting multiple cellular pathways that isolated compounds might miss [17,19].

Similarly to other OLE compounds, they possess a potentiality to induce human’s protective enzyme systems—leading to preventive effects against several degenerative disorders such as diabetes, cancer, and neurodegenerative diseases. In vivo studies revealed that administration of luteolin significantly improved not only the cognitive function in rats suffering from chronic cerebral hypo-perfusion [20], but also showed a preventive effect in mice against cognitive defects induced by a high-fat diet [21]. Furthermore, luteolin may prevent cognitive defects by reducing the formation of β-amyloid plaques, a hallmark of Alzheimer’s disease, by inactivating the enzyme Glycogen Synthase Kinase (GSK)3α, which effectively suppresses the generation of the plaques [17].

Recent research illustrated that also the flavonoid apigenin offers a unique multi-target approach to protect the brain and fighting cancer. It has been highlighted that apigenin is far more than a simple anti-oxidant; it acts as a molecular regulator that can cross the blood–brain barrier to directly influence the health of our neurons [17]. In the context of Alzheimer’s disease, apigenin serves as a comprehensive stabilizer; it may indeed protect neurons from the toxicity of β-amyloid by preserving mitochondrial integrity and preventing the leakage of cellular membranes [22]. Apigenin effectively re-wires the brain’s internal communication lines, by restoring the ERK/CREB/BDNF signaling pathway, which is essential for memory and synaptic health. Furthermore, apigenin functions as a powerful molecule against Parkinson’s disease, since it protects dopaminergic neurons by stopping the formation of toxic α-synuclein clumps and lowering brain-wide inflammation [23].

Similar features are shared by rutin (quercetin rutinoside), which also recently emerged as a senomorphic agent [24]. The authors screened a library of natural medicinal agents and emphasized the emerging value of rutin in targeting human senescent cells. However, further assessments disclosed its functional mechanism and pharmacological value in the modulation of senescence associated phenotypes, suggesting its potential benefits in governing the activities of senescent cells. Beyond this peculiarity, rutin has anti-oxidant properties—reducing different oxidizing species such as superoxide, peroxyl, and hydroxyl radicals provides substantial benefits [25] and exerts anti-microbial and anti-inflammatory functions [26,27].

### 1.4. Phenolic and Hydroxycinnamic Acids

While secoiridoids often take center stage, scientific research reveals that phenolic and hydroxycinnamic acids represent a vital, multi-functional fraction of the OLE chemical orchestra. This category includes bioactive performers such as caffeic acid, verbascoside, ferulic acid, gallic acid, and chlorogenic acid, which work in natural harmony to amplify the body defenses.

It has been reported that the neuroprotective potential of OLE is significantly bolstered by its phenolic acid content. Components, such as *p*-hydroxybenzoic acid, are strongly linked to the superior neuroprotective activity found in Spanish and Italian olive leaves [6]. Specifically, verbascoside and its derivative decaffeoylverbascoside, may act as potent inhibitors of acetylcholinesterase. By preventing the breakdown of acetylcholine, these acids help preserve the communication lines between neurons, offering a natural strategy to combat age-related cognitive decline and Alzheimer’s disease [6]. The same authors highlighted that the recipe of these acids is never static and it shifts based on where the tree grows. While Greek samples are notably rich in quinic acid, Spanish and Italian extracts are distinguished by their high concentrations of hydroxycinnamic acids and flavonoids. These findings suggest that the therapeutic potential of an extract is deeply tied to its geographic origin, which dictates the specific balance of molecular tools available to fight disease and inflammation.

## 2. Anti-Tumor Effects of OLE: Where Do We Stand?

In recent years, OLE attracted a growing interest over time for its biological activities, not only for beneficial effects on wellness, but also for potential anti-cancer use. This is witnessed by the increased numbers of published papers using a Pubmed search and the keywords “Olive leaves extract” or “Olive leaves extract and cancer”. The first search highlights 20 works published in the years 1970–2000, 665 from 2000 and 2020 and 527 from 2020 and 2026. The second search revealed published papers only after the year 2020 and 111 works from 2000 to 2026. However, a more detailed analysis showed that only 68 out of the 111 published papers are focused on the anti-tumor activities of OLE, and some of its components, against human cancers of different origin using cell lines and pre-clinical models.

Nonetheless, an effective anti-tumor strategy should not simply target the cancer cell directly, but it is equally crucial to intervene in the patient’s immune system cells and possibly the microenvironment where the tumor thrives. In this context, OLE may serve as a multi-target agent, capable of operating simultaneously on several biological fronts. Furthermore, OLE may offer a promising approach for cancer treatment, compared to the individual compounds, thus providing novel opportunities for cancer therapy as well as increasing the sensitivity of tumor cells to conventional anticancer therapy.

With this background, we first reviewed the studies addressing the effects of OLE on immune effector cells and, subsequently, recent data highlighting anti-tumor effects in pediatric tumors (i.e., neuroblastoma and acute leukemias), which were reinforced by different research on adult tumors.

## 3. OLE Modulates Immune-Cells with Anti-Tumor Properties

Recent investigations highlighted OLE as a versatile modulator of the human immune system, capable of both suppressing excessive inflammatory responses and strengthening anti-tumor immunity. While OLE contains a complex array of biophenols, its immune-modulatory effects are largely driven by oleuropein and its metabolites, which act as multi-target agents on various immune cell populations.

A primary mechanism driven by OLE involves the reprogramming of the myeloid cell compartment. A recent study [28] demonstrated that oleuropein can transform immune-suppressive cells, such as myeloid-derived suppressor cells (MDSCs) and tumor-associated macrophages, into immune-stimulatory subsets that acquired the characteristics of dendritic cells and M1-like macrophages. This reprogramming is marked by an increase in antigen presentation markers and the production of interleukin (IL)-12, one of the most potent anti-cancer cytokines. Consequently, this shift significantly potentiates systemic T-cell responses and enhances the efficacy of modern immunotherapies involving, for example, the programmed cell death (PD)-1 blockade [28].

In a different experimental model, the authors [10] documented that OLE modulates immune-responses in the context of obesity-associated inflammation by directly modulating macrophage polarization. The authors reported that OLE significantly suppressed pro-inflammatory mediators by reducing the production and gene expression of tumor necrosis factor (TNF)-α, IL-6, and IL-1β as well as nitric oxide, prostaglandin E2, and reactive oxygen species. In parallel, OLE enhanced anti-inflammatory responses by increasing the secretion of IL-10. Importantly, OLE induced a phenotypic switch in macrophages, reprogramming them from an inflammatory state (M1) to a protective one (M2). Regarding the molecular mechanisms, three main signaling pathways were highlighted: (i) the inhibition of nuclear factor kappa-light-chain-enhancer of activated B cells (NF-kB), (ii) the activation of Peroxisome Proliferator-Activated Receptors (PPAR)-γ, a master regulator of the M2 phenotype, and (iii) the nuclear factor erythroid 2-related factor (NRF)2-Kelch-like ECH-Associated Protein (KEAP)-1 pathway, attenuating the oxidative stress [10].

Regarding the activity of OLE on T lymphocytes, evidence suggests a dominant immune-stimulation. It has been observed that OLE treatment increases spleen cellularity in tumor-bearing models, which indicates a reconstitution of the immune system’s overall reactivity [9]. This immune-stimulating effect strongly boosts both the effector and memory functions of tumor-killing CD4^+^ and CD8^+^ T cells. These therapies induce protective systemic tumor-specific T-cell responses that can lead to complete tumor regression [28]. Furthermore, the authors reported that oleuropein treatment leads to a significant increase in CD137^+^CD4^+^ T cells within tumors, a marker associated with enhanced T-cell activation and survival. Furthermore, when combined with anti-PD1 antibodies, oleuropein helps overcome resistance to immunotherapy, leading to an increase in tumor-infiltrating T cells and a reduction in inhibitory markers such as T-cell Immunoglobulin and Mucin domain (TIM)-3 on CD4^+^ lymphocytes [28]. Finally, microarray analysis demonstrated that OLE (specifically the Chemlali variety) modulates the expression of genes involved in the T-cell receptor signaling pathway, such as TNF Receptor-Associated Factor (TRAF)-6. This factor is a positive regulator of IL-12 biosynthetic process, which is a critical driver for the differentiation and activation of T cells toward an anti-tumor phenotype [29].

Another important immune cell population modulated by OLE is represented by NK cells. In the context of cancer, specifically leukemia and melanoma, OLE does not just kill tumor cells directly, but seems to unleash NK cells to more effectively identify and destroy malignant targets through paralleled mechanisms. Treatment with OLE, especially when combined with the cytokine IL-28B, significantly increased the gene expression of perforin and granzyme, thus improving the NK cells’ ability to punch holes in and kill tumor blasts [30]. Notably, OLE and IL-28B are both effective alone, but their combination is far more potent. This dual approach targets multiple cancer-sustaining pathways simultaneously, reducing the likelihood of the tumor developing drug resistance and significantly improving survival rates in animal models [30]. Next, OLE treatment stimulates NK cells to release significantly higher levels of interferon (IFN)-γ that not only helps in direct tumor lysis, but also enhances broad immune responses [9]. Another mechanism is related to the reduction in the serum of Transforming Growth Factor (TGF)-β1, a cytokine down-regulating NK cell activating receptors and reducing their cytolytic activity.

## 4. Neuroblastoma

### 4.1. General Features

Neuroblastoma (NB), the most common extracranial solid tumor of pediatric age, accounts for 15% of cancer-related deaths in children and adolescents. It originates in the developing sympathetic nervous system from neural crest elements. The primary sites of tumor occurrence are the adrenal medulla and the paraspinal ganglia. NB is mostly encountered in children under the age of five, with a median age at diagnosis of 18 months [31]. NB usually arises sporadically, and the main somatic genetic alterations observed include: (i) the amplification of the MYCN oncogene; (ii) segmental chromosomal aberrations such as the gain of 17q, the loss of 1p (1p36), and the loss of 11q (11q23); (iii) the mutation/amplification of the anaplastic lymphoma kinase (ALK) oncogene. Cases of familial NB are observed in 1–2% of NB patients, and mutations in the ALK oncogene account for most of these hereditary cases [32].

From a clinical standpoint, NB presents with broad heterogeneity, with favorable forms of the disease undergoing spontaneous regression, whereas aggressive forms have an unfavorable outcome. The disease presents as metastatic at time of diagnosis in about 50% of patients, with BM and regional lymph nodes being the most common sites of metastases. The International Neuroblastoma Risk Group (INRG) classification clusters NB-affected patients into four different groups referred to as L1 and L2 (loco-regional), M (metastatic), MS (metastatic special) [33]. This classification relies on image-defined risk factors, whose evaluation is carried out before any treatment. Further, based on INRG classification and the integration with several other parameters, such as the age at diagnosis, the amplification of MYCN gene, the tumor cell ploidy, the tumor histology, and the presence of segmental chromosomal aberrations, NB-affected patients can be stratified into low-, intermediate-, and high-risk (HR). The prognosis is favorable for low- and intermediate-risk patients, while unfavorable for the high-risk ones. This patient’s stratification is of pivotal importance because it guides the therapy approaches to be adopted, which broadly span from observation only to intensive and multi-modal therapy for the HR patients [34]. For these latter approaches, the standard of care treatment includes: induction chemotherapy, which uses a combination chemotherapy regimen, followed by surgical resection, high-dose myeloablative chemotherapy with autologous stem cell rescue, radiation therapy, and post-consolidation immunotherapy with anti-GD2 mAbs [31].

In recent decades, most of the research efforts focused on improving the survival rate and quality of life for those patients affected by HR-NB, whose prognosis is still dismal. Indeed, in the application of aggressive and multi-modal treatment regimens, the 5-year survival rate is unsatisfactory—remaining approximately at 50%—thus challenging researchers and clinicians to find new ways for fighting NB. One of the most popular routes, currently, is represented by the advent of precision medicine. Indeed, a lot of energy has been committed to the creation of appropriate programs, whose focus lies on wider patient genome profiling, in order to find out genetic alterations potentially actionable. Examples of these programs focused on pediatric malignancies include the Australian Zero Childhood Cancer (ZERO) [35], the European MAPPYACTS [36], the German INFORM program [37], and the Italian PREME program [38] which instead is specifically committed to NB-patients. Besides the personalized medicine programs, an emerging treatment strategy for relapsed/refractory HR-NB patients is represented by an immunotherapeutic cell-based approach, consisting in the infusion of anti-GD2 chimeric antigen receptor (CAR) T cells. The results of phase I/II clinical study show promising results [39].

### 4.2. OLE Anti-NB Activities

A primary way in which OLE acts against NB is by directly reducing cell viability in a dose- and time-dependent manner. Interestingly, these effects have been validated not only in traditional 2D cell cultures, but also in more sophisticated 3D tumor spheroids, which closely mimic the architecture and treatment resistance of actual solid tumors [40]. The biological sabotage of the tumor begins with a halt in the cell division machinery, as supported by the demonstration that OLE induced a G0/G1 phase cell cycle arrest, by down-regulating growth-promoting genes such as cyclin D1 (CCND1), cyclin-dependent kinases CDK4 and CDK6, and simultaneously boosting the expression of p53 and the cell cycle inhibitor p21 (CDKN1A). Beyond stopping growth, we reported that OLE is highly effective at directly inducing NB cells into apoptosis [40]. The first demonstration was reported by Seçme M. et al. using oleuropein alone [41]. This study highlighted how oleuropein, the main component of OLE, shifts the balance between pro-apoptotic and anti-apoptotic proteins, significantly increasing the expression of genes such as BAX, BAD and BID, while simultaneously reducing the level of the anti-apoptotic protein BCL-2. This molecular imbalance leads to the loss of mitochondrial membrane potential and the subsequent activation of caspases-9 and -3, which act as the primary inducer of apoptosis. The study by our group revealed that OLE triggers this self-destruction through a dual-pathway approach: it engages the extrinsic pathway by activating caspase-8 and the intrinsic (mitochondrial) pathway by increasing the ratio of pro-apoptotic proteins like BAX over anti-apoptotic ones like BCL-2. This leads to the final activation of the executioner enzymes caspases-3 and -7, which induce apoptosis. Furthermore, OLE concomitantly activates the NF-kB pathway through the phosphorylation of the p65 subunit that in NB seems to contribute to the inflammatory and apoptotic response [40].

Another critical factor in inducing apoptosis in NB is the production of reactive oxygen species [42,43]. Treatment with OLE causes an increase in total ROS, which serves as a cytotoxic stress signal, triggering the apoptotic cascade. The combination of these diverse molecular mechanisms, the BAX/BCL-2 imbalance, the activation of caspases-3, -7, -8, and -9, cell cycle arrest, and oxidative stress makes OLE a powerful agent potentially capable of overcoming the resistance of NB cells and inducing their selective elimination. Furthermore, OLE and its derivatives, such as oleacein, have been reported to contribute to NB cell death by inhibiting signaling pathways, such as the transcription factor STAT3, whose inhibition reduces not only survival but also the invasive and metastatic capacity of malignant cells [44,45]. In this context, additional evidence highlights that OLE may serve as a shield against tumor progression by inhibiting cell migration and invasion. The most direct proofs of this activity come from our studies, where we utilized the scratch assays to observe cell movement [40]. In these experiments, a gap or wound is physically created in a cell layer; while untreated cancer cells naturally migrate to fill the void, NB cells treated with OLE (specifically the IMR-32 and SH-SY5Y cell lines) were unable to close the gap. This inhibitory effect was dose-dependent, with the wound remaining significantly wider in treated groups over 24, 36, and 48 h, essentially trapping the cancer cells in place. At the molecular level, such immobilization is driven by the disruption of signals that drive tumor movement.

**Figure 2:**
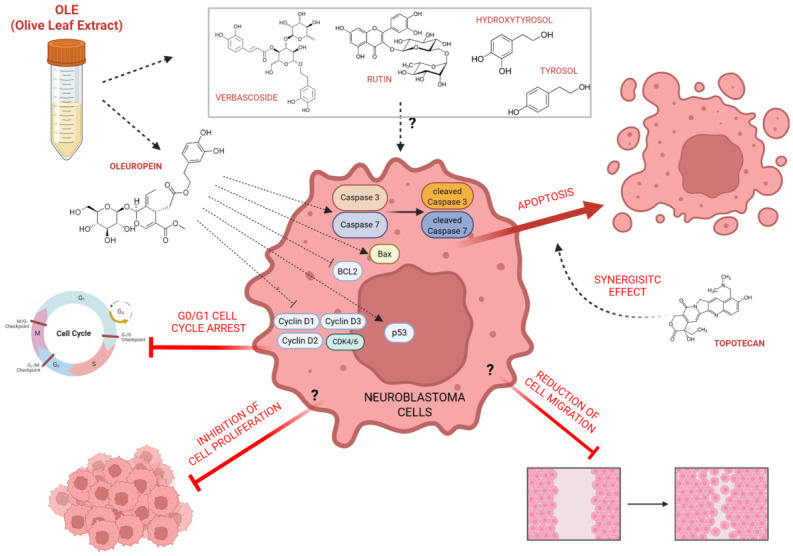
Anti-tumor effects of OLE on NB cells. Created in BioRender. Morandi, F. (2026) https://BioRender.com/6t5v8rf. Accessed on 29 May 2026.

A significant clinical advantage of OLE, as emphasized in our study, is its proven effectiveness in 3D tumor spheroids, which more accurately mimic the complex architecture of NB in patients, compared to traditional 2D cultures [40]. Finally, one of the most promising translational findings is that OLE is able to act as a chemosensitizer. Indeed, we demonstrated that OLE works in powerful synergy with topotecan, a standard chemotherapy drug used for HR-NB patients. This combination significantly enhanced the reduction in tumor cell viability, compared to either agent alone, suggesting that OLE could potentially be used as a safe, aqueous food supplement to improve the therapeutic index of conventional treatments while reducing their harsh side effects in these pediatric patients [46]. Figure 2 describes the mechanisms of action of OLE on NB cells.

## 5. Pediatric Acute Leukemias

Pediatric acute leukemias are aggressive blood cancers that begin when hematopoietic progenitor cells in the BM undergo a malignant transformation and start proliferating uncontrollably. These diseases are characterized by a massive buildup of immature, poorly differentiated cells, known as blasts, which flood the BM and peripheral blood, ultimately dampening the production of healthy blood cells. Statistically, these neoplasms are significant, accounting for roughly 27% to 33% of all childhood cancer diagnoses. Depending on their cellular origin, these leukemias are divided into two main categories that are the Acute Lymphoblastic Leukemia (ALL) and the Acute Myeloid Leukemia (AML). ALL is the most common form of cancer found in children and occurs when lymphoid cell precursors stop maturing at a specific stage and begin to multiply abnormally. Within ALL, two primary groups are defined and include the B-ALL (approximately 80% of cases) and the T-ALL (around 20% of cases). B-ALL involves B-cell progenitors and is highly heterogeneous [46,47,48].

### 5.1. General Features of Pediatric B-ALL

Leukemic blasts in B-ALL patients express the pan-B lineage marker CD19, along with CD10, which defines common ALL subtypes, CD34, terminal deoxynucleotidyl transferase (TdT) and variable levels of CD20 and CD22. Flow cytometric analysis is essential for diagnosis, subtype assignment, and minimal residual disease (MRD) monitoring [49]. Different genetic abnormalities have been detected in leukemic blasts, and some of them are associated with a favorable prognosis. Among them are the fusion gene ETV6/RUNX1—present in 20–25% of all cases and generated by the translocation of chromosomes 12 and 21 (t 12;21)—and high hyperdiploidy (51–67 chromosomes, 25% of cases) [49,50]. In contrast, different genetic abnormalities are associated with high-risk B-ALL, including BCR/ABL1 Philadelphia chromosome-positive ALL (3–5% of pediatric cases), KMT2A rearrangements (prevalent in infants), hypodiploidy (often associated with TP53 alterations), amplification of chromosome 21 and the Philadelphia-like subtype, IKZF1 deletions and TCF3/PBX1 fusion gene caused by t(1;19) [49,50,51,52].

The prognosis of B-ALL patients dramatically improved in recent years, with event-free survival at five years ranging from 88 to 92% and overall survival reaching 95% in standard-risk group [50,53]. However, 15% of B-ALL patients relapsed and displayed a worse prognosis, with only 50% 5-year overall survival [54], although the introduction of immunotherapeutic protocols is starting to improve their outcome [52,54]. In this context, allogeneic stem cell transplantation remains the definitive consolidation therapy for high-risk B-ALL patients who achieve remission, as it provides a lasting graft-versus-leukemia effect. However, exiting results have been obtained also using bispecific T-cell engagers or CD19-directed CAR-T cells. As described by the authors [55], the bispecific T-cell engager blinatumomab effectively directs the immune system to kill CD19^+^ leukemia cells. Thus, blinatumomab is increasingly used as a bridge to transplant because it is highly effective at reaching the critical goal of MRD negativity while having fewer side effects than intensive chemotherapy. In cases of relapse, the same authors reported that combining or sequencing different targeted agents, such as using inotuzumab ozogamicin (which targets CD22) followed by blinatumomab, can help achieve deep remissions before a transplant. For the most difficult cases, Del Bufalo et al. highlighted the success of CAR-T-cell therapy that involves engineering a patient or donor T lymphocytes to recognize and destroy leukemia cells. This approach has revolutionized the treatment of children and young adults with relapsed/refractory B-cell precursor acute lymphoblastic leukemia [56,57]. Nonetheless, novel combined therapies are welcome, not only for relapsed patients, but also for patients undergoing standard chemotherapeutic protocols, in order to limit the toxicity of these treatments. In this view, the use of nutraceuticals may represent a promising tool.

### 5.2. General Features of Pediatric AML

AML is the second most frequent hematological malignancy in the pediatric population and arises from the transformation of myeloid stem or progenitor cells. Its classification has evolved significantly over the years and is based on morphological and genetic abnormalities [58]. While older systems (FAB) categorized AML based on how the cells looked under a microscope (subtypes M0 through M7), modern classification (WHO/ICC) focuses on the molecular alterations. These include rearrangements like KMT2A (very common in infants), mutations in NPM1 or CEBPA, and Acute Promyelocytic Leukemia (APL) defined by the PML-RARA fusion. Furthermore, there are unique subgroups such as the myeloid leukemia associated with Down Syndrome and secondary myeloid neoplasms that occur after previous cytotoxic treatments. To provide the best possible care, patients may be stratified into risk groups (i.e., standard, intermediate, high, or very-high risk). This ensures that the intensity of the treatment matches the aggressiveness of the disease. Stratification is guided by three main factors including (i) the clinical presentation, such as the patient age and initial white blood cell count, (ii) the presence of genetic anomalies, like nucleoporin (NUP)98 translocations or the core-binding factor (CBF) A2T3:GLIS2 mutation, immediately signal a high-risk status, and (iii) MRD. In the 1980s, the vast majority of children with AML did not survive, but today, five-year survival rates have risen to 65–75% [59,60]. Survival is closely tied to how a patient is stratified. In the recent Italian AIEOP-AML-2013 protocol, 3-year survival rates were 97% for standard risk patients (a result now comparable to ALL), 84.2% for intermediate risk and 79.4% for high-risk [61]. Nonetheless, certain genetic forms still remain very difficult to cure, such as those with CBFA2T3:GLIS2 or FUS:ERG fusions, where survival can drop below 40%. Furthermore, relapse remains the primary cause of treatment failure, occurring in 30–40% of cases. For these children, a second transplant can be a life-saving option, with recent data showing a 3-year survival rate of around 37.5% [60]. The treatment for AML is generally more intensive than for B-ALL and relies on a combination of strong chemotherapy and newer molecularly targeted drugs. In this context, Locatelli et al. report on the Italian AIEOP-AML-2013 trial, which used ICE (idarubicin, cytarabine, and etoposide) as a standard first course [61]. They also tested FLA-My (fludarabine, cytarabine, and liposomal doxorubicin) as a second induction course and found that both regimens were equally effective in helping patients reach complete remission [61]. Allogeneic stem cell transplantation is actually considered the standard consolidation treatment for any child classified as high-risk in their first remission. The authors [62] highlighted that achieving morphological remission before transplant is one of the most important factors for long-term success. However, recent studies reported the efficacy of combined treatment with gemtuzumab-ozogamicin, an anti-CD33 agent, and chemotherapy for children whose leukemia cells express the CD33 antigen, as well as the use of FLT3 inhibitors like sorafenib for children with the FLT3-ITD mutation [58]. These authors also highlighted the promise of venetoclax (a BCL2 inhibitor) and menin inhibitors (like revumenib) for treating aggressive forms like KMT2A-rearranged AML. Thus, these can be used to stabilize the disease before a transplant or as maintenance therapy afterward. In pediatric AML, innovative cellular therapies have been explored including engineered Tr1 cells (LV-10), which have shown the potential to kill AML blasts while avoiding the dangerous complication of graft-*versus*-host disease [63] or CAR T and NK cells [64,65,66,67].

### 5.3. OLE Anti-Tumor Activities Against Pediatric Acute Leukemias

The first detailed study on the anti-tumor activities of OLE against acute pediatric leukemias, alone and in combination with conventional chemotherapeutic agents, has been recently reported by our group [12]. Previous evidence was provided exclusively using human promyelocytic leukemia HL-60 cells [68,69,70] and the myeloid K562 cell line [29,71]. Using the HL-60 cells, it has been documented that OLE, oleuropein and luteolin exerted a cytotoxic effect in a dose-dependent manner and that the mechanism underlying was related to the apoptotic pathway, with DNA laddering and cytoplasmic and nuclear changes [69]. The principal bioactive phenols in OLE have been identified and quantified, and the authors emphasized the extract anti-genotoxic effect, showing its ability to protect the DNA of healthy cells from oxidative damage induced by hydrogen peroxide. Crescimanno M. and coworkers analyzed the effects of olive oil phenols on HL-60 cell line that was either sensitive or resistant to anthracyclines [70], whereas Abaza L. and colleagues reported that OLE (specifically from the Tunisian Gerboui variety) inhibited cell growth and induced differentiation into the granulocyte lineage, identifying apigenin 7-glucoside as the primary bioactive constituent responsible for this maturation process [68].

Regarding the K562 cells, OLE (specifically the Chemlali variety) halted cell proliferation and induced apoptosis and monocyte/macrophage differentiation by modulating specific genes such as IFI16 and early growth response protein (EGR)-1 [29]. An additional anti-tumor effect was reported, demonstrating that OLE can counteract the Warburg effect through the reduction in the glycolysis rate by downregulating critical glucose and lactate transporters (i.e., GLUT1 and MCT4), essentially starving the malignant cells of their energy source [71].

**Figure 3:**
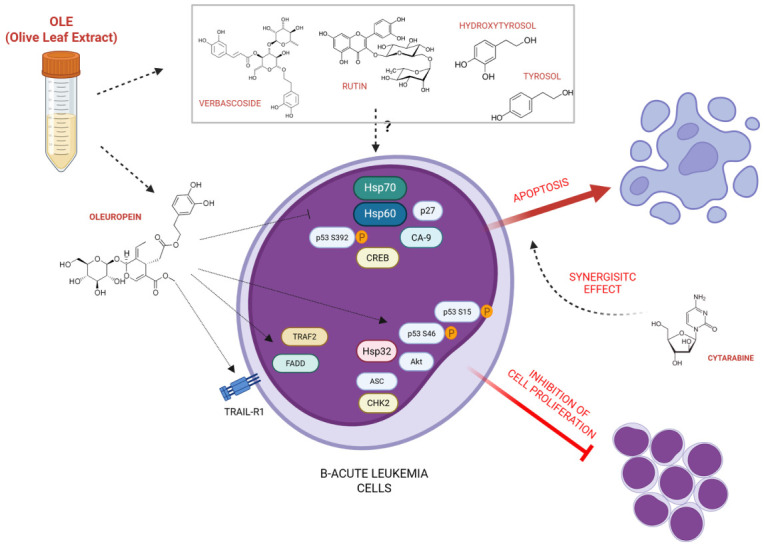
Anti-tumor effects of OLE on B-ALL cells. Created in BioRender. Morandi, F. (2026) https://BioRender.com/0zujg5e. Accessed on 29 May 2026.

In our recent work, we investigated the anti-tumor effects of OLE in vitro against human acute leukemia and lymphoma cells using not only cell lines, but also primary blasts from patients [12]. We clearly documented OLE as a specific natural weapon against B-ALL, highlighting that B-ALL cells possess a selective vulnerability to the extract compared to other malignancies such as AML or certain lymphomas. OLE successfully induced apoptosis in primary leukemic blasts collected from patients, both at diagnosis and at relapse, suggesting that it may bypass some of the resistance mechanisms that tumors develop after standard treatment. By contrast, such an effect was observed only marginally in primary AML blasts. Interestingly, some proteins were modulated in opposite ways in B-ALL and AML, potentially explaining their different response to OLE. At molecular level, we documented that OLE triggered the extrinsic apoptotic pathway by increasing the expression of the Tumor necrosis factor-Related Apoptosis-Inducing Ligand (TRAIL) receptor-1 and its supporting proteins, Fas-Associated Death Domain (FADD) and TRAF2. Simultaneously, OLE activated CHK2—a critical protein that signals DNA damage—and modulated the master tumor suppressor p53, by increasing its phosphorylation at specific sites (S15 and S46) needed to activate death-inducing genes. Furthermore, OLE treatment led to a drastic reduction in protective proteins such as carbonic anhydrase-9, survival factors like CREB, and various heat-shock proteins (HSP-27, HSP-60, and HSP-70) that normally help cancer cells to survive stress and resist therapy. To understand whether OLE may be effective in combination with chemotherapy in B-ALL, we selected the two standards of care agents, cytarabine and cyclophosphamide. In particular, cytarabine is currently used to treat patients with relapse/refractory disease, whereas cyclophosphamide represents a standard of care treatment for B-ALL patients. We highlighted a powerful synergic/additive proapoptotic effect of OLE with cytarabine, but not with cyclophosphamide; thus, we supposed that the different effects achieved could result from the different molecular mechanisms of action of the two chemotherapeutic agents. Indeed, cyclophosphamide acts as an alkylating agent, interfering with DNA replication and transcription of mRNA. In contrast, cytarabine induces DNA damage through the incorporation of similar pyrimidine and purine nucleoside, leading to the activation of two kinase pathways such as ATR-CHK1 and ATM-CHK2, which may lead to DNA repair of apoptosis. Due to the finding that OLE up-regulated CHK2 in ALL (but not in AML) cell lines, we speculated that the synergistic/additive effects observed with cytarabine could be related to the concomitant induction of CHK2 in target cells (Figure 3).

Such evidence, together with the knowledge that OLE is, at least to the current knowledge, free of toxicity [72], allowed us to emphasize that the olive leaf extract could represent an ideal adjuvant candidate to improve the therapeutic outcomes for B-ALL children that can be easily administered as a food supplement, as approved by regulatory agencies worldwide (EMA/94696/2017, FDA GRN 001119). However, the lack of specific studies on pediatric subjects and lack of data regarding possible interactions with chemotherapy must be taken into account.

The principal findings obtained from studies in vitro using OLE on cell lines of pediatric tumors or primary cells from pediatric patients are summarized in Table 1.

## 6. Additional Evidence of OLE Activities: Studies on Adult Tumors

Current knowledge describes OLE as a versatile biological compound that employs a multi-front strategy against solid tumors. Unlike conventional therapies that often target a single pathway, OLE contains a complex of chemical components, notably oleuropein and HT, that work synergistically to dampen cancer survival, spreading, and metabolism while leaving healthy cells unharmed. The mechanisms underlying this are related to different pathways including cell cycle arrest, induction of apoptosis, modulation of invasiveness and spreading, reduction in angiogenesis and cell metabolisms.

In the case of breast cancer, which remains a major global health challenge, OLE has shown exceptional versatility. Two studies [73,74] demonstrated that oleuropein significantly inhibits the viability of both estrogen receptor-positive (MCF-7) and more aggressive triple-negative breast cancer cells (MDA-MB-231). A particularly fascinating discovery revealed that HT can actually complex with copper within triple-negative breast cancer cells, reducing their aggressiveness and metastatic potential [75]. Furthermore, OLE acts as a powerful sensitizer, allowing conventional chemotherapies like paclitaxel or doxorubicin to work more effectively at lower, less toxic doses.

Melanoma, one of the most treatment-refractory skin cancers, is another major target for OLE. It has been documented that oleuropein is not merely an anti-oxidant but a potent anti-tumor agent capable of targeting human melanoma cells (A375 line) harboring the BRAF mutation, which are often highly resistant to conventional treatments [76]. These studies demonstrated that while high doses of oleuropein (500 µM) directly stimulate apoptosis, more moderate doses (250 µM) can effectively slow the proliferation of malignant cells by acting on the pAKT/pS6 signaling pathway. Importantly, it has been emphasized that whole OLE is often more effective than isolated oleuropein and that the extract may act in synergy with conventional chemotherapeutic drugs. In this regard, it has been reported that OLE significantly enhanced the anti-tumor activities of dacarbazine and everolimus, even successfully targeting melanoma cells that have developed resistance to standard BRAF inhibitors such as vemurafenib [76].

In more recent studies, the same group explored a new frontier: the metabolic sabotage of the tumor [71]. They demonstrated that OLE is able to counteract the Warburg effect, which is the dependency of tumors on aerobic glycolysis to produce energy. The extract drastically reduced the glycolytic rate of tumor cells by decreasing the expression of key proteins, such as the glucose transporter (GLUT)-1, the enzyme PKM2, and the lactate transporter MCT4. Furthermore, it was pointed out that this metabolic blockade was not limited to melanoma, but also confirmed in colon carcinoma, breast cancer, and chronic myeloid leukemia. Finally, the authors provided evidence of the safety profile of OLE, noting that at concentrations effective against tumors, the extract did not modify the viability of healthy cells, such as human mesenchymal stromal cells. The latter finding supported the concept that OLE is an ideal candidate nutraceutical for integrated and complementary oncological therapies [71].

The reach of OLE extended deep into the central nervous system as well. It was reported that OLE employs a multi-targeted strategy to dismantle the survival mechanisms of glioblastoma multiforme (GBM), one of the most aggressive and treatment-resistant brain tumors [77]. One of the primary ways OLE fights GBM is by starving the tumor and blocking its ability to spread. Tezcan G. and coworkers documented that OLE significantly reduces tumor weight and vascularization by inhibiting vascular endothelial growth factor (VEGF)-A, but also Matrix Metallo-Proteinase (MMP)-2 and MMP-9. A particularly significant finding was that OLE, similarly to that reported in other tumors, worked in powerful synergy with bevacizumab, a standard anti-angiogenic drug, making the treatment far more effective at reducing tumor mass than the drug alone [77]. An additional anti-tumor mechanism was the inhibition of the Akt signaling pathway [78]. By blocking the phosphorylation of Akt and increasing the ratio of pro-apoptotic proteins like BCL-2-associated X protein (BAX) over anti-apoptotic ones, like blocks programmed cell death (BCL)-2, OLE effectively induced apoptosis. Another fascinating layer of OLE activity was its ability to modulate microRNA (e.g., miR-137, miR-145, miR-153, and Let-7d) expression in tumor cells that affected the induction of apoptotic pathways [77,79]. Of note, OLE was also able to sensitize tumor cells to temozolomide in GBM.

In gastrointestinal cancers, oleuropein not only effectively curtailed the growth and survival of these malignant cells—in a manner that was both dose- and time-dependent—but could also suppress the epithelial–mesenchymal transition, blocking migration and metastasis. Indeed, the molecule forced early apoptosis, by inducing caspase-3 while simultaneously silencing BCL-2, and down-regulated invasive markers such as vimentin, SNAIL1, and MMP-13. At the same time, it restored the expression of E-cadherin, which helped keep cells fixed in their healthy epithelial state [80].

The anti-tumor mechanisms of OLE and their components described above have also been reported for additional solid tumors such as colorectal cancer [81,82], hepatocellular carcinoma [83], cervical cancer [84], seminoma [85] and osteosarcoma [86,87].

Regarding the role of OLE components against hematological tumors, most of the studies have been focused on multiple myeloma (MM), a malignancy characterized by the accumulation of clonal plasma cells in the bone marrow (BM). One of the most critical breakthroughs in this field involves the synthetic olive derivative oleyl HT and the identification of the interferon regulatory factor (IRF)4–c-MYC axis as a vital “Achilles’ heel” for myeloma cells [88]. Oleyl HT was found to significantly down-regulate the expression of IRF4, a master transcription factor that myeloma cells depend on for survival. Consequently, MM cells were inhibited in their growth. Furthermore, a severe endoplasmic reticulum stress was observed and unfolded protein response activated, leading to apoptosis through the activation of genes like caspase-10 and Blimp-1.

A major challenge in treating MM is the protection that cancer cells receive from the BM stromal cells, which often shield them from conventional chemotherapy. Todoerti K. and coworkers also demonstrated that oleyl HT is uniquely capable of penetrating this defense, successfully reducing the viability of myeloma cells even when they are hidden and co-cultured with protective stromal cells [88].

Beyond killing the MM cells, Leto G. et al. highlighted that OLE addresses the debilitating bone loss and fractures that are hallmarks of the disease [15]. Indeed, oleuropein possesses powerful bone-anabolic and osteo-protective effects. It recalibrates the BM environment by promoting the differentiation of mesenchymal stem cells into osteoblasts and simultaneously inhibiting osteoclastogenesis. This dual action makes OLE a promising candidate for maintaining skeletal integrity in myeloma patients.

Finally, the clinical potential of these compounds is bolstered by their selective cytotoxicity. Two studies [15,88] both emphasized that while these olive derivatives impact on the growth of malignant plasma cells, they remain completely non-toxic to healthy human cells, such as peripheral blood mononuclear cells, B lymphocytes, and mesenchymal stem cells. This high safety profile positions OLE as a robust potential adjuvant for enhancing the efficacy of conventional treatments, while protecting healthy tissues.

## 7. Conclusions and Future Perspectives

The future of OLE in oncology is shifting from its traditional role as a dietary staple to becoming a sophisticated nutraceutical in integrated cancer care. Scientific perspectives suggest that OLE and its derivatives, like oleuropein and HT, are poised to transform from laboratory interests into powerful clinical tools, particularly as non-toxic adjuvants.

The most immediate perspective for OLE lies in its use as a nutraceutical adjuvant to enhance conventional chemotherapy while shielding healthy tissues. In the field of pediatric tumors, OLE may be used in synergy with cytarabine to kill B-ALL cells, potentially allowing clinicians to achieve similar or better results with fewer toxic doses of chemotherapy. Similar synergistic potential may be envisaged in NB, where OLE could be used effectively with topotecan. Furthermore, we planned to also test OLE in vitro on pediatric T-ALL, since the effects of other nutraceuticals have already been demonstrated on this type of leukemia [89].

Notably, an issue that may require additional investigation is that regarding the bioavailability. Although some individual components (i.e., oleuropein and HT) are absorbed and cleared relatively quickly, OLE in liquid form results in peak oleuropein levels that are six times higher than taking the same dose in capsules. This means that the formulation should be optimized in order to achieve the best clinical benefit. To fix this, scientists are developing nanotechnology solutions, such as liposomes or nanoparticles, which may act like a protective delivery van to keep the OLE stable in the blood longer and improve its concentration in tumor tissues. Others are creating semi-synthetic derivatives that are more lipophilic (fat-soluble), allowing them to slip through cell membranes more easily.

Finally, one of the most valuable attributes of OLE is its selective cytotoxicity and safety. Indeed, while OLE and its phenols impact on the growth of various cancer types through different and parallel mechanisms and protect healthy tissues from damage induced by chemotherapy and radiation. Because OLE is a safe, water-soluble nutraceutical, it can be easily administered orally, making it an ideal candidate for long-term supportive care—especially in pediatric patients.

## Figures and Tables

**Table 1:** Effects of total olive leaf extract in vitro on pediatric tumor models.

Tumor Type	Experimental Model	Oleuropein (μM)	Exposure Time	Main Biological Effects	Molecular Pathways	Combination with Chemotherapeutic Agents
**Neuroblastoma**	2D and 3D in vitro culture of cell lines	50–300	48–120 h	Reduction of cell viability; Inhibition of cell proliferation; Cell cycle arrest; Induction of apoptosis; Inhibition of cell migration;	increased phospho-NFkb increased phosppho-Bcl2 increased cleaved caspases 3 and 7	Synergy with Topotecan
**AML**	Cell lines and primary blasts from patients	50–400	24–72 h	Inhibition of cell proliferation; Induction of apoptosis; (only at the highest concentration)	Reduction of TRAIL R1, CD40, NEMO and TRAF, modest modulation of apoptotic pathway and protein phosphorylation	none
**B-ALL**	Cell lines and primary blasts from patients	50–400	24–72 h	Inhibition of cell proliferation; Induction of apoptosis;	Reduction of CA9, HSP60, HSP70, p27, SOD2 (cell stress pathway), increase of TRAF2 and ASC/CARD5 (NFkb pathway), increase of TRAIL R1, HSP32, clapsin, phospho p53 S15 (apoptosis pathway), increased phosphorylation of Akt, c-jun, p53 S46, CHK2 and decreased phosphorylation of p53 S392 (phosphorylation pathway)	Synergy with cytarabine, no effect with cyclophosphamide

## Data Availability

No new data were created or analyzed in this study.

## Abbreviations

The following abbreviations are used in this manuscript:

OLE	Olive leaf extract
HT	hydroxytyrosol
GSK	Glycogen Synthase Kinase
MDSCs	myeloid-derived suppressor cells
PD	programmed cell death
TNF	tumor necrosis factor
IL	interleukin
NF-kB	nuclear factor kappa-light-chain-enhancer of activated B cells
PPARs	Peroxisome proliferator-activated receptors
NRF	nuclear factor erythroid 2-related factor
KEAP	Kelch-like ECH-associated protein
TIM	T cell immunoglobulin and mucin domain
TRAF	TNF receptor associated factors
IFN	interferon
TGF	transforming growth factor
GLUT	glucose transporter
GBM	glioblastoma multiforme
VEGF	vascular endothelial growth factor
MMP	matrix metalloproteinase
BCL	blocks programmed cell death
BAX	Bcl-2–associated X protein
MM	multiple myeloma
BM	bone marrow
IRF	interferon regulatory factor
CCND1	cyclin D1
CDK	cyclin-dependent kinases
NB	neuroblastoma
STAT	signal transducer and activator of transcription
EGR	early growth response protein
B-ALL	B-acute lymphoblastic leukemia
AML	acute myeloid leukemia
CA-9	Carbonic Anhydrase 9
HSP	heat shock protein
TRAIL	Tumor necrosis factor-related apoptosis-inducing ligand
FADD	Fas-associated death domain
CAR	Chimeric antigen receptor
ALK	Anaplastic lymphoma kinase
INRG	International Neuroblastoma Risk Group
HR	High-risk
